# Separate and Combined Response to UV-B Radiation and Jasmonic Acid on Photosynthesis and Growth Characteristics of *Scutellaria baicalensis*

**DOI:** 10.3390/ijms19041194

**Published:** 2018-04-13

**Authors:** Jiaxin Quan, Shanshan Song, Kadir Abdulrashid, Yongfu Chai, Ming Yue, Xiao Liu

**Affiliations:** 1 Key Laboratory of Resource Biology and Biotechnology in Western China, Ministry of Education, Northwest University, Xi’an 710069, China; 18740422231@163.com (J.Q.); songss333@163.com (S.S.); kadir_ks@126.com (K.A.); chaiyfhappy@163.com (Y.C.); nwuyueming@163.com (M.Y.); 2 College of Life and Geography Sciences, Kashgar University, Kashgar 844006, China

**Keywords:** UV-B, jasmonic acid, chlorophyll fluorescence, gas exchange, root system architecture

## Abstract

The negative effects of enhanced ultraviolet-B (UV-B) on plant growth and development have been reported with many species. Considering the ability of jasmonic acid (JA) to improve plant stress tolerance, the hypothesis that JA pretreatment could alleviate the adverse effects of UV-B on *S. baicalensis* was tested in this study with photosynthesis and growth characteristics. The results showed that UV-B or JA alone both induced photosynthesis inhibition and decreased biomass in stems and leaves. However, the photosynthetic reduction caused by increased UV-B was mainly related to the effect of nonstomatal-limitation, while that of JA was a stomatal-limitation effect. JA pretreatment prior to UV-B could remit the photosynthetic inhibition via the recovery of chlorophyll content, stomatal conductance; and intercellular CO_2_ concentration (especially the maximum electron transport rate increase). Furthermore, the coaction of JA and enhanced UV-B alleviated some disadvantageous effects on the leaf and did not aggravate the growth damage induced by their separate actions.

## 1. Introduction

*Scutellaria baicalensis* Georgi is a species of perennial plant belonging to the Lamiaceae and is one of the most important medicinal herbs. It is officially listed in the pharmacopoeias of many countries to help anti-inflammatory, anticancer, antibacterial activities, and so on [1,2]. Flavonoid compounds are the main active ingredient and are regarded as the quality evaluation index of *S. baicalensis*; therefore, researchers are always looking for ways to improve the efficacy of *S. baicalensis* by increasing the flavonoid level.

Ultraviolet-B (UV-B) radiation, as a small portion of the solar spectrum, has a disproportionately large photobiological effect due to its absorption by important biological molecules [3]. Furthermore, UV-B radiation has significant effects on plant secondary metabolism, especially, accumulation of flavonoid compounds, which is regarded as one of the most significant protective responses against UV-B radiation. Flavonoids can filter UV-B radiation before it reaches sensitive molecules in mesophyll cells and cause oxidative degradation of membrane lipids [4,5,6]. However, the negative effects of enhanced UV-B on plant growth and development have been reported in many research studies. These studies showed that UV-B caused damage to DNA, proteins and membranes and impeded photosynthetic activities and plant growth [7].

Jasmonic acid (JA), the simplest nontraditional plant hormone, has diverse effects and functions in regulating plant developmental processes [8]. Furthermore, as reported for other hormones, exogenous application of JAs can induce the tolerance of plants to various stresses [9,10,11,12,13,14]. Researchers of stressed plants showed that exogenous JA or methyl jasmonate (MeJA) could block the stress-induced photosynthetic inhibitory effect by the alleviation of leaf CO_2_ assimilation, reducing salinity stress [15] and the relief of photosynthetic rate depression caused by paraquat or NaCl stress [16,17,18]. Moreover, the role of exogenous JAs in counteracting UV-B stress has been confirmed in Kentucky bluegrass, barley and wheat seedlings by the index of the antioxidant system, photosynthetic pigments and basic fluorescence [19,20,21].

Our previous field work proved that *S. baicalensis* was sensitive to elevated UV-B radiation [1,22]. Then, could exogenous JA remit the damaging effect of increased UV-B on the growth of *S. baicalensis*? And what were the effects of coaction of JA and UV-B radiation? In this study, we focused on the interactive effect of UV-B radiation and exogenous JA on the photosynthesis and growth characteristics of *S. baicalensis*. The aim was to ascertain the effects of separate and combined response of enhanced UV-B radiation and JA and UV-B on *S. baicalensis*.

## 2. Results

### 2.1. Light-Response Curves

**Figure 1:**
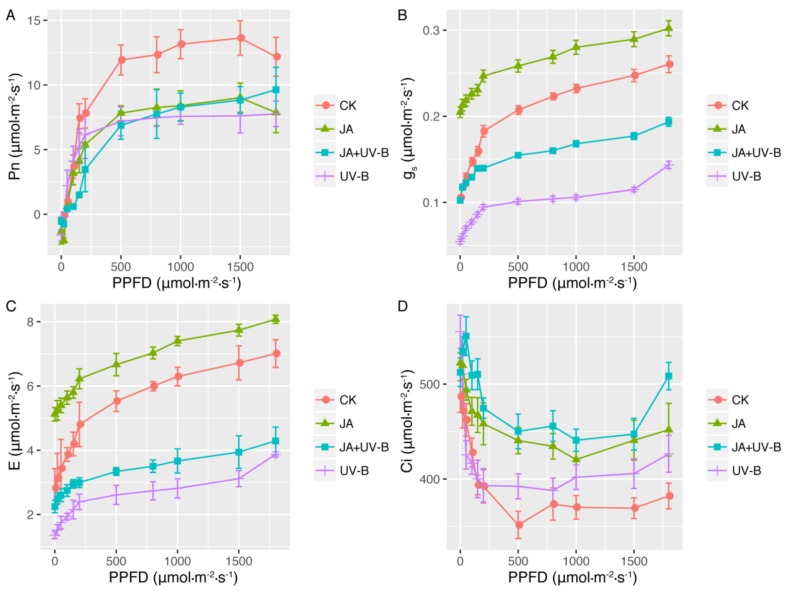
The curves of the response of net photosynthetic rate (Pn) (**A**), stomatal conductance (gs) (**B**), transpiration rate (E) (**C**) and intercellular CO_2_ concentration (Ci) (**D**) to photosynthetic photon flux density (PPFD) for *Scutellaria baicalensis* Georgi under different treatments. CK (the control), JA (seedlings were sprayed with 1 mM jasmonic acid (JA)), UV-B (seedlings were treated with 15-d elevated ultraviolet-B (UV-B) radiation), JA+UV-B (seedlings were sprayed with 1 mM JA before additional 15-d UV-B radiation). Measurements were made from 0 to 1800 μmol·m^−2^·s^−1^. Each point represents the mean value of 3 leaves from 3 seedlings. Data are means ± S.E.

For the light-response curves of the gas exchange measured, the net photosynthetic rate (Pn) of all groups increased in a curve-linear fashion with increasing photosynthetic photon flux density (PPFD); the shapes of the curves, however, differed among the groups (Figure 1A). Compared to the control, both JA treatments (JA and JA+UV-B groups) and UV-B radiation (UV-B group) caused a decrease in P_n_ and the light-saturated maximum photosynthesis (P_max_) values. The values of gs and E increased with increasing PPFD in all groups. Among the four groups, the JA group had the highest gs and E, control (CK) had the second highest, followed by the JA+UV-B and UV-B groups, and that of the JA+UV-B group was higher than that of the UV-B group (Figure 1B,C). The C_i_ value decreased rapidly at a lower PPFD and increased gradually with increasing PPFD in all groups. Compared to CK, the JA (JA and JA+UV-B groups) and UV-B treatments reduced the value of C_i_, while the JA+UV-B group showed the highest values (Figure 1D).

The fitting results of the photosynthetic light-response curves are shown in Table 1. The greatest decrease in P_max_ was observed in the JA and UV-B groups (38.27% and 34.46%, respectively), and the pretreatment of a UV-B-stressed seedling with JA remitted the decrease (24.01%). For light compensation point (LCP) and light saturation point (LSP) values, the UV-B group had no significant difference from the control, but the values of the JA and JA+UV-B groups increased significantly. For LSP and LCP the JA and JA+UV-B groups had higher values than those of the CK and UV-B groups (*p* < 0.05). Compared to the CK group, R_d_ increased 96.08% in the UV-B and 62.75% in the JA groups, the JA+UV-B group had the minimum value, and the JA group had the maximum. Ф increased 60% in the UV-B group but decreased 60% in the JA+UV-B group. CK owned the maximum value of θ, the UV-B group had the minimum, and the JA and JA+UV-B groups had similar θ values (Table 1).

### 2.2. Chlorophyll Fluorescence

The values of F_0_, F_m_ and F_v_/F_m_ of the different groups are also presented in Table 1. Compared to the CK group, F_0_ decreased under the JA treatment significantly, and the UV-B group showed higher F_0_ and F_m_ values than those of the JA and JA+UV-B groups. However, the F_v_/F_m_ of the four groups did not show a significant difference.

**Figure 2:**
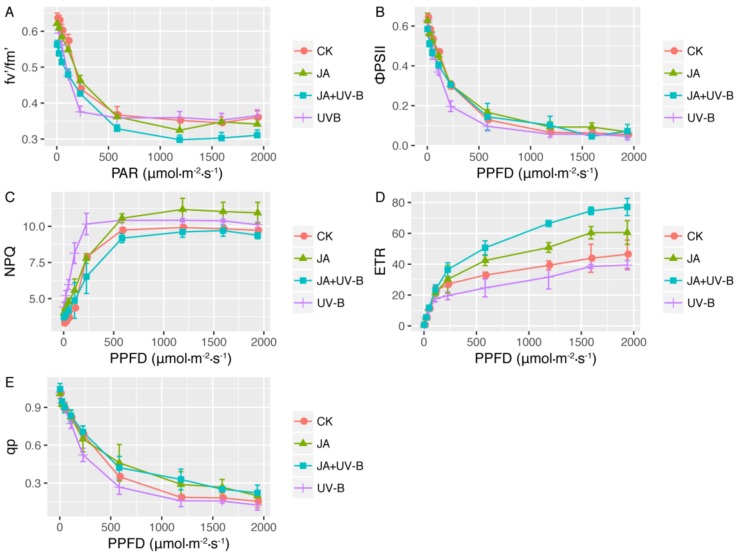
Influence of UV-B radiation and JA pretreatment on electron transport rate (ETR) (**D**), photochemical quenching (qp) (**E**), non-photochemical quenching (NPQ) (**C**), actual PSII efficiency (ФPSII) (**B**) and efficiency of energy harvested by open PSII reaction centers (Fv’/Fm’) (**A**) from light-response curves of fluorescence in different treatments. CK (the control), JA (seedlings were sprayed with 1 mM JA), UV-B (seedlings were treated with 15-d elevated UV-B radiation), JA+UV-B (seedlings were sprayed with 1 mM JA before additional 15-d UV-B radiation). Measurements were made from 0 to 1950 μmol·m^−2^·s^−1^. Each point represents the mean value of 3 leaves from 3 seedlings. Data are means ± S.E.

The light-response curves of chlorophyll fluorescence are shown in Figure 2 and indicate that the values of qp, ФPSII and F_v_’/F_m_’ all decreased, while the electron transport rate (ETR) and non-photochemical quenching (NPQ) increased with increasing PPFD. The maximum ETR derived from the curves increased progressively in the JA and JA+UV-B groups, and the JA+UV-B treatment had the highest value. Furthermore, at low PPFD, there was little difference in F_v_’/F_m_’ among the four groups, but when PPFD increased, the decrease in F_v_’/F_m_’ was great in the JA+UV-B groups, and the JA+UV-B group had the minimum value. The UV-B group had the minimum ФPSII value, and the q_p_ value of the JA group was lower than that of the other three groups. UV-B radiation (UV-B and JA+UV-B groups) promoted q_p_, especially at a higher PPFD. The JA treatment improved NPQ significantly at whole PPFD, but the promotion caused by UV-B radiation showed only at a higher PPFD. JA+UV-B and CK had similar values of NPQ at whole PPFD (Figure 2). The ETR_max_ value was not influenced by UV-B radiation, but increased in the JA and JA+UV-B groups, and the JA+UV-B showed the highest ETR_max_ (Table 1).

### 2.3. Chloroplast Pigment Content

JA, UV-B, and their combined treatment all did not show significant effects on Car content; however, they had a different influence on chlorophyll (Table 2). For example, although the JA treatment did not affect the content of Chl *b*, it decreased Chl *a* content, and thus caused a reduction in Chl *(a + b)* and Chl *a/b* content. UV-B radiation reduced the content of Chl *a*, Chl *b*, and the total chlorophyll concentration. Compared to CK, the combination of UV-B and JA treatment only lowered the concentration of Chl *a/b*, which relieved the depression of total chlorophyll content caused by the JA or UV-B treatment.

### 2.4. Growth Parameters and Phenotyping Analysis of Root System Architecture

Table 3 shows the analytical results of the growth and phenotyping parameters of the roots. The JA treatment decreased the seedling height and total biomass, and the decrease in biomass was mainly caused by the reduction in aboveground biomass (including leaves and stem). Although root biomass was not affected by the JA treatment, root surface area and average root diameter decreased. UV-B radiation did not have a significant effect on height, but it reduced the biomass of the leaves and stem, which led to a total biomass decrease. In addition, UV-B radiation caused an increase in leaf area but a reduction in the specific leaf area (SLA) value. It was found that more parameters were depressed in the JA+UV-B group, such as seedling height, total biomass, total root length, root surface area and average root diameter. For the JA+UV-B group, the decrease in total biomass was related to the stem and roots, and not the leaves.

## 3. Discussion

### 3.1. The Effects of JA and UV-B on Photosynthesis

In our study, photosynthesis of *S. baicalensis* was depressed by UV-B radiation, and the deleterious effects of UV-B radiation in our study was consistent with many other studies that had documented that increased UV-B could result in a 3–90% decrease in photosynthesis of different plants due to direct (effect on photosystem) and indirect (decrease in pigment and leaf area) effects [23,24]. In this study, the photosynthesis inhibition of the UV-B radiation was by nonstomatal limitation, which led to the decrease in gs and E subsequently. In addition, the inhibition effect was mainly a result of the decrease in ETR and Chl *a* and *b* content. The slowing of ETR could be due to the damage of electron transfer components induced by the UV-B radiation. For example, Q_A_^-^ was photosensitive, and primary quinone was bound to the D_2_ protein, so photosensitized plastosemiquinones by UV-B radiation may destroy the D2 protein directly and in turn inhibit the electron transport of PS II [25]. Moreover, there was a concern that with a reduction in electron transport capacity by enhanced UV-B other effects such as a reduction in *Cytf* content, damage of the PS I protein environment, lower ATPase and photophosphorylation activities would ensue, and all of these could cause a modification in the transformation efficiency of electric energy into active chemical energy [26,27,28,29].

Moreover, the decrease in photosynthetic pigment by UV-B radiation in our study was also a concern in regards to the inhibition of their synthesis or degradation. Especially, the selective destruction of Chl *a* biosynthesis or degradation of its precursors, which leads to deleterious effects on light-harvest and photochemical reactions [30], resulting in the restriction of photosynthesis.

Although a negative effect of UV-B on net photosynthetic rate was observed, the seedlings of *S. baicalensis* developed some strategies to adapt to the UV-B radiated environment. For instance, the capability of using low light was promoted, which might be related to the increase in NPQ. The increase in NPQ and the decrease in ETR in our study were consistent with the results of beech seedlings, sessile oak and wheat under conditions of enhanced UV-B radiation [21,31,32]. The improved NPQ increased thermal energy dissipation by regulating the antenna system to reduce the damage to the photosynthetic system under UV-B radiation, which is regarded as an effective defensive mechanism of plants under a stress environment [33,34].

The negative role of JA on photosynthesis had been observed more than 20 years ago when down-regulation of rubisco by JA was identified in barley [35], and it was further revealed in *Brassica napus* L., soybean and other plants [16,36,37,38]. The seedlings of *S. baicalensis* under JA treatment alone also exhibited some detrimental effects on photosynthesis, similar to those seen if treated with UV-B radiation alone. For instance, it also induced a reduction in the P_n_ and P_max_, but it differed from the UV-B radiated seedlings, and the g_s_, E, and C_i_ values improved under JA. Thus, stomatal limitation was one of the most important factors of photosynthetic inhibition under the JA treatment condition. JA induced stomatal closure via ABA was proven by Raghavendra and Reddy in 1987, and the reduction in stomatal conductance inhibited CO_2_ absorption and ultimately resulted in photosynthetic activity decline [39,40]. Furthermore, in our experiment, photosynthesis impediment by JA was connected to the reduction in Chl *a* and Chl *a/b* content. Many researchers have reported that the chlorophyll breakdown induced by JA and MeJA and the loss of photosynthetic pigments decreased the amount of energy absorbed by the photosynthetic apparatus, thereby attenuating energy requiring anabolic events [16,17,37,38,41]. Meanwhile, a reduction in Chl *a/b* content by JA in our results exhibited that the fold domain of the photosynthetic membrane decreased, which reduced the light energy transfer efficiency. The JA-treated-seedlings had the highest NPQ in the low-intensity light, which suggested that energy dissipation increased under the low-light conditions. The increase in NPQ and F_0_ caused by JA implied enhancement of thermal dissipation in the antenna system, which also led to photosynthesis inhibition. In contrast to the UV-B radiated-seedlings, increased LCP and LSP values indicated the utilization of higher light of the JA treated-seedlings improved.

After the co-action of JA and UV-B, compared to the control group, although P_max_ was depressed, the value was higher than that of the separate JA or UV-B treatments, which meant JA pretreatment could alleviate the damage caused by subsequent UV-B radiation. The remission of photosynthesis was related to the recovery of total chlorophyll content, g_s_, and C_i_, especially, and the highest ETR. Although photosynthetic inhibited effects of JA have been reported, the ameliorating effects on stressed plants by pretreated JA has also been proven, similar to the results of our study [16,17]. However, there have been some opposite results of exogenous JAs to stress-induced photosynthetic capability. For instance, an impaired effect caused by the MeJA pretreatment on gas-exchange attributes was reported in drought-stressed soybean [37]. Therefore, the physiological role of exogenous JAs on photosynthesis is complex, and needs to be investigated using more species under various environments.

### 3.2. The Effects of JA and UV-B on Growth

The injury of photosynthetic capacity caused by enhanced UV-B affects plant growth and development as has been reported by numerous researchers [23,24]. In our study, in addition to the suppression of photosynthesis, the enhancement of respiration resulted in biomass loss of *S. baicalensis* under UV-B radiation conditions, and the loss was caused by the decrease in stem and leaf biomass. The decrease in total leaf biomass was a result of less leaves. In addition, the SLA decrease by UV-B indicated the increase in leaf thickness or leaf mass density. Lower SLA is regarded as a common strategy for plants living under stressed environments [42]. This suggested that UV-B-radiated *S. baicalensis* allocated less biomass to the light-stressed aboveground part by defoliation, and improved the tolerance of the remaining leaves by increasing the sclerenchyma or lignin level. Additionally, UV-B radiation had no significant influence on root biomass, but it increased root surface area and average root diameter, which benefited the absorption of underground resources.

The restraint of photosynthesis, along with the rise in respiration under the JA alone treatment, led to the reduction in aboveground biomass of *S. baicalensis* seedlings. Similar to UV-B radiation, JA also reduced the stem and leaf biomass, and the leaf mass loss was also correlated with leaf falling, which had been proven by others and was linked to downregulation of housekeeping proteins encoded by photosynthetic genes and upregulation of genes active in defense reactions against stresses [8]. Furthermore, the seedling height decreased under the JA treatment but not under UV-B radiation. The growth-related inhibitory effect of JA in our study has also been reported for soybean, *Rumex obtusifolius*, Scots pine, rice and so on [37,43,44], which was due to the inhibition effect of JAs through the JA receptor COI1 by reducing both cell number and size [45].

The effects of JAs on stressed-plant growth are discrepant; some reports have exhibited that exogenous JAs could improve the resistance to salinity stress, drought stress, chilling injury, UV-B radiation and heat stress by increasing antioxidant enzyme activities, enhancing water potential, lowering membrane permeability, increasing unsaturated fatty acid content, and other means [21,46]. However, some results have found otherwise; for example, the growth-inhibitory effect of MeJA treatment was more pronounced in drought-stressed soybean, which decreased the plant height, the node height, leaf area, stem diameter, number of nodes on main stem, branches and so on [37]. In our study, unlike that of JA or UV-B treatment, leaf biomass of *S. baicalensis* was not affected after the co-action of JA and UV-B radiation. The biomass decrease by coaction was mainly related to the stem and roots, especially a root biomass reduction, which was only observed in the co-acted seedling. Our results showed that the combined effects of JA and UV-B had more similar effects to those of JA alone, such as the effect on seedling height, biomass per leaf, leaf area, SLA, root surface area and average root diameter, which were significantly different from those of UV-B. In addition, the co-action did not worsen the effects on other growth indexes compared to the UV-B alone or JA treatments. It further remitted the negative effects on decreased leaf biomass by JA or UV-B, and the SLA decrease and root biomass increase by UV-B.

## 4. Materials and Methods

### 4.1. Plant Material

The experiment was carried out in the spring of 2016. The seeds of *Scutellaria baicalensis* Georgi were collected from medicine market of Shangluo area of Shaanxi. After surface sterilization for 30 min using 0.01% HgCl_2_, uniform-sized and vital seeds were rinsed three times in sterile water and then sown in a glass culture dish for germination. Germinant seeds were rooted in plastic pots (13-cm-diameter, 12-cm-tall) filled with a soil mixture of 25% sand, 25% organic matter, and 50% peat in a greenhouse at the campus of Northwest University in Xi’an (latitude 34.3° N, longitude 108.9° E, altitude 397 m). The pots were placed under white fluorescent lamps (400 µmol m^−2^ s^−1^) with a 14/10 h light/dark cycle, 35% relative humidity and 25/18 °C day/night temperature cycle. The environment of pots was controlled by greenhouse auto control system. To minimize the effect of microenvironment variation, the position of pots was changed daily.

A month later, uniform seedlings approximately 15 cm in height were divided into four groups: CK, UV-B, JA, and JA+UV-B treatments. Each treatment contained 15 seedlings. Details of the different treatments in the experiment are listed in Table 4.

### 4.2. JA Treatment

The seedlings of the JA treatment group were sprayed with 1 mM JA (Sigma-Aldrich Co., St. Louis, MO, USA) as performed in Liu et al. [21]. The volume of JA was uniform for each treatment. To avoid treatment contamination, treatments were sprayed independently outside the culture room, and plants were left until complete evaporation of the sprayed solution. Then, one-half of the seedlings were subjected to additional UV-B radiation.

### 4.3. UV-B Treatment

UV-B radiation was artificially supplied by square-wave UV-B fluorescent lamps (36 W, Beijing Lighting Research Institute, Beijing, China) following the procedure described in Liu et al. (2012) [21]. The maximum output of these lamps was 313 nm. The lamps were suspended above the plants and were switched on for 8 h per day from 9:00 to 17:00 for 15 days. The lamps were wrapped with either 0.13-mm cellulose acetate film (Grafix Plastics, Cleveland, OH, transmission down to 290 nm) for the supplemental UV-B radiation group (UV-B group) or with 0.13-mm polyester plastic film (Grafix Plastics, Cleveland, OH, USA, absorbs radiation below 320 nm) for the control group. The enhanced UV-B radiation (10.30 kJ·m^−2^·d^−1^) at the top of the plant canopy was adjusted by altering the distance from the lamps to the canopy.

The amount of UV-B radiation was measured using a UV radiometer (Handy, Beijing, China) every second day. The cellulose acetate and polyester plastic films were replaced every 5 days.

Immediately after the end of the UV-B radiation, the young and most fully expanded healthy leaves from 3 to 5 plants were collected for subsequent measurements.

### 4.4. Light-Response Curves of Photosynthesis

Gas exchange measurements were made on intact leaves using an open gas exchange system, LI-6400 photosynthesis (LI-COR Inc., Lincoln, NE, USA). Artificial illumination was applied to the leaves from a red-blue LED light source. P_n_, gs, C_i_, and E were measured at different levels of PPFD, and the PPFD was from 0 to 1800 μmol·m^−2^·s^−1^ in 11 steps (0, 20, 50, 100, 150, 200, 500, 800, 1000, 1500, and 1800 μmol·m^−2^·s^−1^). The light-response curves of the photosynthesis curves were fitted using a nonrectangular hyperbola least square curve fitting procedure [47] as described in Equation (1), where Ф was the apparent quantum efficiency, Q was the PAR, P_max_ was the light saturated rate of CO_2_ assimilation, *θ* was the convexity or curvature factor, R_d_ was the dark respiration, and P was the photosynthetic rate. During all measurements, CO_2_ concentration inside the reference chamber was set to 400 μmol·mol^–1^, the leaf block was set at 25 °C, and the flow rate of air was 400 μmol·s^–1^. LSP and LCP were calculated using the linear regressions of PPFD (0–200 μmol·m^−2^·s^−1^) and net photosynthetic rate curve.

(1) $$P=\frac{\Phi Q+P_{\max}-\sqrt{{\left(\Phi Q+P_{\max}\right)}^{2}-4\Phi Q\theta P_{\max}}}{2\theta }-R_{d}$$

### 4.5. Chlorophyll Fluorescence Parameters

The measurement of chlorophyll fluorescence parameters was performed using intact leaves using an LI-6400-40 leaf chamber fluorometer (LCF) attached to the LI-6400 photosynthesis system, which provided LED-based fluorescence and a light source. After at least 30 min of adaptation to the dark, the minimal fluorescence yield (F_0_) was determined by measuring the modulated light that was sufficiently low so as not to induce any significant variable fluorescence, and the maximal fluorescence yield (F_m_) was determined using a 0.8-s saturating pulse at 6000 μmol m^−2^ s^−1^ in the dark-adapted leaves. The steady-state value of fluorescence (F_s_) was recorded with light-adapted leaves, and a saturating pulse at 6000 μmol m^−2^·s^−1^ was imposed to determine the maximal fluorescence level (F_m_’). In both dark- and light-adapted states, the fluorescence parameters were expressed using the following formulae [48]: (1) the maximal efficiency of photosystem II (PSII) photochemistry, F_v_/F_m_ = (F_m_ − F_0_)/F_m_; (2) the quantum efficiency of photosystem II, ФPSII = (F_m_’ − F_s_)/F_m_’; (3) the light-adapted efficiency of energy harvested by open PSII reaction centers, F_v_’/F_m_’ = (F_m_’ − F_o_’)/F_m_’; (4) the photosynthetic ETR was estimated after Faraloni et al. (2011) [49] by multiplying ФPSII × incident PPFD by 0.5 (two photons were used for exciting one electron because it was assumed there was an equal distribution of excitation between PSII and PSI), and by 0.84, which was considered the most common leaf absorbance coefficient for C3 plants, ETR = (F_m_’ − F_s_)/F_m_’ × PPFD × 0.5 × 0.84; (5) the photochemical quenching, q_P_ = (F_m_’ − F’_s_)/(F_m_’ − F_0′_); and (6) theNPQ was calculated using the expression NPQ = (F_m_ − F_m_’)/F_m_’.

The measurement of the light-response curves of the chlorophyll fluorescence started with light-adapted leaves. The parameters of F_v_’/F_m_’, ФPSII, q_P_, NPQ and ETR were measured at different levels of PPFD from 0 to 1800 μmol·m^−2^ ·s^−1^ in 10 steps (0, 20, 50, 100, 200, 500, 1200, 1600, 1800, and 1950 μmol m^−2^ s^−1^). Dynamic analysis of chlorophyll fluorescence was performed using dark-adapted leaves, and the measurements were done under an actinic light at an intensity of 800 μmol·m^−2^ ·s^−1^, by means of application of 10 sequential saturating light pulses at a 3 min interval. The maximum value of ETR (ETR_max_) was calculated by fitting the exponential rise to maximum function to the corresponding ETR with the PPFD curve by using the following equation: Y = a (1 − e^−bx^) + c, where Y was the electron transport rate and x was PPFD; ETRmax was calculated as (a + c) [23].

### 4.6. Measurement of Photosynthetic Pigments

Photosynthetic pigments were extracted using 80% acetone, and absorbance at 470, 663 and 647 nm was measured using a spectrophotometer. The concentration of photosynthetic pigments was determined using the method of Lichtenthaler (1987) [50].

### 4.7. Measurement of Growth Parameters

Seedling height, leaf area and the biomass of leaf, stem and roots were measured at the end of the experiment. A picture of each fresh leaf surface was taken with a digital camera and leaf surface area was measured using Motic Images Plus 2.0 (Motic Instruments Inc., Richmond, BC, Canada). All samples (leaf, stem, and roots) were placed in a drying oven for 72 h at 80 °C, then the dry mass was measured immediately using an electronic balance (SartoriusBT25S, Beijing, China). The specific leaf area (SLA) was calculated as the ratio of leaf area to leaf dry mass.

### 4.8. Phenotyping Analysis of Root System Architecture

Three to five root samples of each treatment were washed carefully from the soil on a 0.5-mm mesh screen after removal of the shoots. After removing debris, an intact root sample was evenly spread apart in a water layer on a transparent tray and imaged at a resolution of 200 dpi (dots per inch) with an Epson scanning system (Epson J221A; Seiko Epson Corp., Nagano, Japan). Root images were analyzed for total root length, root surface area and average root diameter using the WinRHIZO software (V5.0, Regent Instruments, Quebec, QC, Canada).

### 4.9. Statistical Analysis

All of the measurements were performed with at least 3–5 uniform leaves belong to different 3–5 seedlings, and the means and calculated standard error (S.E.) were reported. Prior to analyses, all variables were tested for normality and homoscedasticity of variance. A two-way analysis of variance (ANOVA) and Duncan’s multiple-range test were performed to investigate the effects of exogenous JA and UV-B on the parameters of the seedlings at 0.05 probability levels using STATISTICA 6.0 software (StatSoft Inc., Tulsa, OK, USA).

## 5. Conclusions

Separate and combined effects of JA and UV-B on *S. baicalensis* were studied with photosynthesis and growth characteristics in our research. The P_n_ and P_max_ were both depressed by separate UV-B or JA treatment but with a different mechanism. The photosynthesis inhibition under UV-B was a nonstomatal limitation effect, but the effect under JA was due to stomatal limitation. Furthermore, UV-B radiation increased the low-light utilization capacity of seedlings, while the JA treatment increased high-light utilization. JA pretreatment could alleviate the damage on photosynthesis caused by subsequent UV-B radiation, which was related to the recovery of total chlorophyll content, gs, and Ci, especially, the increase in ETR.

Photosynthesis restraint and respiration enhancement resulted in a total biomass loss for *S. baicalensis* under UV-B radiation or JA treatment, and the loss was caused by stem and leaf biomass decrease. However, the total biomass loss under coaction of JA and UV-B was mainly related to the stem and roots, and leaf biomass was not affected. In general, the coaction did not result in further damage of the growth indexes than that of separate actions. It did remit the decrease in leaf biomass caused by JA or UV-B, and the SLA increase under UV-B.

## Figures and Tables

**Table 1:** JA and UV-B radiation effects on photosynthetic light response curves parameters and fluorescence parameters in *Scutellaria baicalensis* Georgi. leaf respiration (R_d_), apparent quantum efficiency (Ф), light saturated maximum photosynthesis (P_max_), convexity (θ), light compensation point (LCP), light saturation point (LSP), the minimal fluorescence yield (F_0_), the maximal fluorescence yield (Fm), the maximal efficiency of photosystem II (PSII) photochemistry (F_v_/F_m_) and The maximum value of photosynthetic electron transport rate (ETR_max_). CK (the control), JA (seedlings were sprayed with 1 mM JA), UV-B (seedlings were treated with 15-d elevated UV-B radiation), JA+UV-B (seedlings were sprayed with 1 mM JA before additional 15-d UV-B radiation). Data are means ± S.E., error bars are means of three replicates. Different lowercase letters are significantly different at *p* < 0.05 according to Duncan’s multiple range test.

Treatments	R_d_ [μmol^−2^·s^−1^]	Ф [μmolCO_2_·μmol^−1^photon]	Pmax [μmol^−2^·s^−1^]	θ	LCP [μmol^−2^·s^−1^]	LSP [μmol^−2^·s^−1^]	F_o_	F_m_	F_v_/F_m_	ETR_max_
CK	1.02 ± 0.09 ^c^	0.05 ± 0.003 ^b^	14.16 ± 0.12 ^a^	0.91 ± 0.04 ^a^	17.56 ± 0.89 ^b^	364.65 ± 23.29 ^c^	164.50 ± 18.54 ^ab^	790.19 ± 52.22 ^ab^	0.79 ± 0.01 ^a^	41.76 ± 4.87 ^c^
UV-B	1.66 ± 0.08 ^b^	0.08 ± 0.007 ^a^	9.28 ± 0.09 ^c^	0.74 ± 0.03 ^c^	16.93 ± 0.91 ^b^	425.34 ± 19.89 ^c^	203.22 ± 25.26 ^a^	953.93 ± 111.51 ^a^	0.79 ± 0.00 ^a^	37.86 ± 3.34 ^c^
JA	2.00 ± 0.11 ^a^	0.05 ± 0.000 ^b^	8.74 ± 0.90 ^c^	0.83 ± 0.03 ^b^	42.66 ± 1.21 ^a^	601.48 ± 25.28 ^b^	119.13 ±18.08 ^c^	551.60 ± 123.55 ^b^	0.78 ± 0.02 ^a^	58.22 ± 3.21 ^b^
JA+UV-B	0.89 ± 0.05 ^c^	0.02 ± 0.003 ^c^	10.76 ± 0.96 ^b^	0.82 ± 0.06 ^b^	44.79 ± 1.90 ^a^	1042.69 ± 59.68 ^a^	124.86 ± 28.09 ^b c^	475.76 ± 23.46 ^b^	0.78 ± 0.01 ^a^	75.12 ± 5.12 ^a^

**Table 2:** JA and UV-B radiation effects on photosynthesis pigments of *Scutellaria baicalensis* Georgi. chlorophyll a: Chl *a*, chlorophyll *b*: Chl *b*, total chlorophyll: Chl (*a + b*), carotenoid: Car. CK (the control), JA (seedlings were sprayed with 1 mM JA), UV-B (seedlings were treated with 15-d elevated UV-B radiation), JA+UV-B (seedlings were sprayed with 1 mM JA before additional 15-d UV-B radiation). Data are means ± S.E., error bars are means of three replicates. Different lowercase letters are significantly different at *p* < 0.05 according to Duncan’s multiple range test.

Photosynthesis Pigments	CK	JA	UV-B	JA+UV-B
Chl *a* [mg/g]	10.06 ± 1.15 ^a^	6.56 ± 0.75 ^c^	8.19 ± 0.89 ^b^	8.98 ± 0.68 ^a,b^
Chl *b* [mg/g]	3.88 ± 0.24 ^a^	3.79 ± 0.34 ^a^	2.83 ± 0.22 ^b^	4.04 ± 0.16 ^a^
Chl (*a + b*) [mg/g]	13.72 ± 0.27 ^a^	11.99 ± 1.77 ^b^	11.41 ± 1.66 ^b^	12.03 ± 2.31 ^a^
Car [mg/g]	4.30 ± 0.49 ^a^	4.06 ± 0.56 ^a^	3.72 ± 0.22 ^a^	3.93 ± 0.17 ^a^
Chl *a/b* [mg/g]	2.69 ± 0.65 ^a^	2.21 ± 0.24 ^b^	2.72 ± 0.09 ^a^	2.26 ± 0.19 ^b^

**Table 3:** JA and UV-B radiation effects on growth parameters and phenotyping analysis of root system architecture of *Scutellaria baicalensis* Georgi. CK (the control), JA (seedlings were sprayed with 1 mM JA), UV-B (seedlings were treated with 15-d elevated UV-B radiation), JA+UV-B (seedlings were sprayed with 1 mM JA before additional 15-d UV-B radiation). Data are means ± S.E., error bars are means of three replicates. Different lowercase letters are significantly different at *p* < 0.05 according to Duncan’s multiple range test.

Characters	CK	JA	UV-B	JA+UV-B
Seedling height [cm]	18.88 ± 1.88 ^a^	16.13 ± 2.36 ^b^	19.89 ± 2.78 ^a^	15.84 ± 2.12 ^b^
Total biomass [g]	0.18 ± 0.02 ^a^	0.12 ± 0.02 ^b^	0.14 ± 0.03 ^b^	0.13 ± 0.02 ^b^
Roots biomass [g]	0.10 ± 0.03 ^a^	0.07 ± 0.007 ^a^	0.08 ± 0.007 ^a^	0.06 ± 0.006 ^b^
Stem biomass [g]	0.02 ± 0.005 ^a^	0.01 ± 0.002 ^b^	0.01 ± 0.003 ^b^	0.02 ± 0.003 ^b^
Leaf biomass/each plant [g]	0.06 ± 0.008 ^a^	0.03 ± 0.008 ^b^	0.03 ± 0.008 ^b^	0.04 ± 0.007 ^ab^
Biomass/each leaf [g]	0.005 ± 0.0006 ^b^	0.004 ± 0.0009 ^b^	0.006 ± 0.0008 ^a^	0.005 ± 0.0006 ^b^
Dry mass of above-ground [g]	0.08 ± 0.01 ^a^	0.05 ± 0.001 ^b^	0.036 ± 0.004 ^b^	0.05 ± 0.006 ^b^
Leaf area [cm^2^]	1. 30 ± 0.15 ^b^	1.19 ± 0.20 ^b^	1.59 ± 0.18 ^a^	1.25 ± 0.19 ^b^
Specific leaf area (SLA) [cm^2^/g]	236.16 ± 21.26 ^a^	225.75 ± 38.62 ^a^	205.55 ± 6.95 ^b^	234.83 ± 20.97 ^a^
Total root length [cm]	318.51 ± 30.51 ^ab^	241.93 ± 85.38 ^ab^	326.29 ± 37.75 ^a^	185.14 ± 20.47 ^b^
Root surface area [cm^2^]	27.22 ± 8.48 ^b^	25.83 ± 9.56 ^b^	46.72 ± 2.74 ^a^	22.46 ± 3.89 ^b^
Average root diameter [mm]	0.37 ± 0.05 ^b^	0.34 ± 0.02 ^b^	0.47 ± 0.02 ^a^	0.37 ± 0.02 ^b^

**Table 4:** The different treatments conducted in the study.

Treatments	Interpretation
CK	Control
UV-B	Seedlings were treated with 15 days of UV-B radiation
JA	Seedlings were sprayed with 1 mM JA
JA+UV-B	Seedlings were sprayed with 1 mM JA before additional 15 days of UV-B
